# Effect of Rho–Associated Kinase Inhibitor on Growth Behaviors of Human Induced Pluripotent Stem Cells in Suspension Culture

**DOI:** 10.3390/bioengineering9110613

**Published:** 2022-10-25

**Authors:** Takaki Matsumoto, Mee-Hae Kim, Masahiro Kino-oka

**Affiliations:** 1 Department of Biotechnology, Graduate School of Engineering, Osaka University, 2-1 Yamadaoka, Suita 565-0871, Osaka, Japan; 2 Research Base for Cell Manufacturability, Graduate School of Engineering, Osaka University, 2-1 Yamadaoka, Suita 565-0871, Osaka, Japan

**Keywords:** human induced pluripotent stem cell, suspension culture, Rho–associated kinase inhibitor, aggregate formation, growth behavior, collagen type I

## Abstract

Rho–associated protein kinase (ROCK) inhibitors are used for the survival of single-dissociated human induced pluripotent stem cells (hiPSCs); however, their effects on the growth behaviors of hiPSCs in suspension culture are unexplored. Therefore, we investigated the effect of ROCK inhibitor on growth behaviors of two hiPSC lines (Tic and 1383D2) with different formation of aggregate that attached between single cells in suspension culture. The apparent specific growth rate by long-term exposure to Y-27632, a ROCK inhibitor, was maintained throughout the culture. Long-term exposure to ROCK inhibitor led to an increase in cell division throughout the culture in both lines. Immunofluorescence staining confirmed that hiPSCs forming spherical aggregates showed localization of collagen type I on its periphery. In addition, phosphorylated myosin (pMLC) was localized at the periphery in culture under short-term exposure to ROCK inhibitor, whereas pMLC was not detected at whole the aggregate in culture under long-term exposure. Scanning electron microscopy indicated that long-term exposure to ROCK inhibitor blocked the structural alteration on the surface of cell aggregates. These results indicate that pMLC inhibition by long-term ROCK inhibition leads to enhanced growth abilities of hiPSCs in suspension culture by maintaining the structures of extracellular matrices.

## 1. Introduction

Human pluripotent stem cells (hPSCs), which include both human induced pluripo-tent stem cells (hiPSCs) and human embryonic stem cells (hESCs), are powerful tools in clinical and industrial applications because they have the ability of self-renewal and plu-ripotency [1,2]. Therefore, a large number of hPSCs is required for this purpose, and culture techniques are being studied for the efficient production of hPSCs. Suspension culture is suitable for the mass culture of hPSCs because it saves space, has a high density, and is easier to scale up than adhesion culture [3,4]. However, cell growth varies depending on the combination of cell lines, reagents, and culture systems [5,6,7,8]. For large number of cells to be obtained at low cost [9], it is desirable to design a culture process that can suppress such variation and provide a stable supply. Therefore, basic knowledge on how changes in output variables, such as cell aggregate formation, respond to the combination of input variables described above, and identifying the factors that govern such changes, are necessary. Additionally, achieving stable hPSC expansion in suspension culture and establishing an efficient and reproducible culture strategy are essential.

The Rho–associated protein kinase (ROCK) pathway induces cellular apoptosis through myosin hyperactivation, and ROCK inhibitor promotes cell aggregation by suppressing apoptosis in suspension culture [10,11,12]. ROCK, an effector of Rho A, regulates cell proliferation and migration by regulating cadherin and integrin-derived actin at cell adhesion [13]. Because cell connections are important factors in cell aggregate after aggregation, the role of the Rho-ROCK pathway in cell adhesion has been well studied [14]. In this context, ROCK inhibitors have shown beneficial effects in various cell cultures, promoted wound healing and cell survival, and maintained undifferentiated potential in PSCs in adherent culture [15,16]. ROCK inhibitors change the cytoskeleton by the loss of actomyosin, thereby leading to the proliferation and migration of several cell types in adherent culture [17,18,19,20]. ROCK inhibitors induce the survival of hPSCs by enhancing and continuing cell-cell adhesion, promoting cell propagation, maintaining pluripotency, and reducing the dissociation-induced apoptosis of single cell in suspension culture [5,10,15,16]. However, the correlation between ROCK inhibitor-mediated cytoskeletal reorganization, extracellular matrix (ECM) remodeling, and cell growth behavior, has not been elucidated in suspension culture.

The main objective of the research is to understand the effects of ROCK inhibitor on the growth behavior of hiPSC aggregates after cell aggregation. To accomplish this objective, two different cell line (Tic and 1383D2 cell line) with a different character of aggregate formation, are used. In the source of these cell lines, Tic cell line derived human fetus lung cells of a 14-week-old male, and 1383D2 cell line derived from peripheral blood of 36-year-old male [21]. In aggregate formation among two cell lines, it suggested that the large spherical cell aggregate is formed by high production of cell-secreted ECM in suspension culture [5]. We investigated the role of ROCK pathway in mediating the effects of ROCK inhibitor on the growth behavior in aggregates of two different hiPSC lines in suspension culture.

## 2. Materials and Methods

### 2.1. Culture Conditions of hiPSCs

The hiPSC lines (Tic and 1383D2) were obtained from the Japanese Collection of Research Bioresources Cell Bank and the Center for iPS Cell Research and Application at the Kyoto University [21], respectively. hiPSCs were cultured on polystyrene plates coated with laminin-511 E8 fragments (iMatrix-511; Nippi, Inc., Tokyo, Japan) in specific medium (StemFit AK02N medium; Ajinomoto, Tokyo, Japan). Single cells were seeded as 7.5 × 10^3^ cells/cm^2^ with 10 μM ROCK inhibitor (CultureSure Y-27632; Fujifilm Wako Pure Chemical Corp., Osaka, Japan) at 37 °C in a humidified atmosphere with 5% CO_2_. The culture medium was changed every 24 h.

### 2.2. hiPSC Aggregate Culture

After cell detachment from plates on day 4 following the procedure reported in our previous study [22], single cells were seeded at a concentration of 1.0 × 10^5^ cells/mL in a 30-mL stirred suspension bioreactor (BWV-S03A, Able Co., Tokyo, Japan) in AK02N with 10 μM CultureSure Y-27632. The agitation of the bioreactor was controlled at a rotational speed of 55 rpm and the bioreactor was set at 37 °C in a humidified atmosphere [23]. The culture medium was renewed every 24 h. The general culture method that hiPSCs were maintained with Y-27632 for 24 h was defined as short-term exposure to ROCK inhibitor to prevent apoptosis of single-dissociated hiPSCs [5,21]. In contrast, the method in which hiPSCs were maintained with Y-27632 for 120 h was defined as long-term exposure to ROCK inhibitor.

For other experiments, cells were cultured as described above and treated with 10 μM CultureSure Y-27632, 10 μM Fasudil (Calbiochem, La Jolla, CA, USA), and 10 μM Thiazovivin (ReproCELL, Inc., Kanagawa, Japan). In all cases, the ROCK inhibitors were prepared as a concentrated stock solution in sterilized water, with the same volume of sterilized water added to the controls.

### 2.3. Kinetic Analysis of Growth Behavior of hiPSCs

To calculate cell number, cell aggregates were collected at different culture times (*t* = 24, 72, and 120 h). Aggregates were washed with phosphate-buffered saline (PBS) and dissociated into single cells using Accumax (Innovative Cell Technologies Inc., San Diego, CA, USA) with 10 μM ROCK inhibitor. Cells were counted using an automatic cell counter (TC20, Bio-Rad Inc., Hercules, CA, USA). 

To elucidate the kinetics of growth behaviors in aggregates in suspension culture, the apparent specific growth rate, $\mu^{\mathrm{app}}$ (h^−1^), and apparent specific rate of aggregate number, $\eta_{\mathrm{ag}}{}^{\mathrm{app}}$ (h^−1^), were calculated as follows using cell density, *X* (cells/mL) at culture time, *t* (h) and aggregate number, *n*_a_ (aggregates):

(1) $$\mu^{\mathrm{app}}=\frac{1}{X_{t}}\cdot \frac{{dX}_{t}}{dt}$$

(2) $$\eta_{\mathrm{ag}}^{\mathrm{app}}=\frac{1}{n_{a}}\cdot \frac{{dn}_{a}}{dt}$$

The growth processes of hiPSC aggregates in suspension culture were evaluated based on the apparent specific growth rate and apparent specific decreasing rate of aggregates, as previously described [8].

(3) $$\mu^{\mathrm{app}}{=\mu }_{g}-{\;k}_{d}$$

(4) $$\eta_{\mathrm{ag}}^{\mathrm{app}}{=k}_{\mathrm{bu}}-{\;k}_{\mathrm{coa}}$$

where *µ*_g_ is the specific growth rate (h^−1^), *k*_d_ is the specific death rate (h^−1^), and *k*_bu_ and *k*_coa_ are the specific break-up and coalescent rates of cell aggregates (h^−1^).

The projected area of cell aggregates, *S* (μm^2^) were measured after collection of cell aggregates at 24, 72, and 120 h, as previously described [8]. In three independent experiments, 500 μL of medium with cell aggregate was obtained from a 30 mL bioreactor and bright-field images of whole cell aggregates within the medium were captured using image analyzer (IN Cell Analyzer 2000; GE Healthcare, Chicago, IL, USA) at 24, 72, and 120 h. Cell aggregates with sizes greater than *S* = 3 × 10^2^ μm^2^ were counted. The three experiments were summed for all conditions to make the distribution of the projected area of cell aggregates.

### 2.4. Time-Lapse Observation

To understand coalescence between the aggregates in the two hiPSC lines, we monitored the coalescence process between two aggregates in static suspension culture using a time-lapse microscope (BioStudio-T; Nikon Engineering, Co., Ltd., Kanagawa, Japan) equipped with a camera for video imaging. Briefly, the cells were resuspended in a medium containing 10 μM ROCK inhibitor in low-attachment 96-well V-bottom plates (Prime Surface 96 V Plate; Sumitomo Bakelite Co., Ltd., Tokyo, Japan) at 2.0 × 10^2^ cells/well. Cell aggregates were collected at 72 h and reseeded onto low-attachment 96-well V-bottom plates. Coalescence between aggregates was observed using a 4× objective lens, and images were obtained every 20 min for 100 min at several positions.

### 2.5. Preparation and Staining of Frozen Sections

The immunofluorescence staining of frozen sections was performed as previously described [5,6,7,8]. Frozen cell aggregates were cut into 10-μm thick sections of using a cryostat (Leica CM1850; Leica, Wetzlar, Germany). Aggregates were thaw-mounted onto adhesive glass slides (Matsunami Glass Ind., Ltd., Osaka, Japan). After soaking for 5 min in PBS containing 0.25% Triton X-100, nonspecific proteins were blocked using Block Ace (Dainippon Sumitomo Pharma Co., Ltd., Osaka, Japan) for 1 h. The slides were incubated with primary antibodies anti-collagen type I (Abcam, Cambridge, MA, USA) and anti-phosphorylated myosin light chain (pMLC) 2 (Ser19) mouse (Cell Signaling Technology Inc., Beverly, MA, USA) overnight at 4 °C. The aggregates were washed with Tris-buffered saline (TBS) and immunolabeled with Alexa Fluor 488-conjugated goat anti-rabbit or anti-mouse antibodies (Life Technologies, Grand Island, NY, USA) for 1 h. Cell nuclei and F-actin were stained with 4′,6-diamidino-2-phenylindole (DAPI; Life Technologies) and rhodamine phalloidin (Thermo Fisher Scientific, Waltham, MA, USA), respectively. Images were obtained using a confocal laser scanning microscope (FV1000; Olympus, Tokyo, Japan) with a 60× objective lens and image analyzer under fluorescence excitation at 488–594 nm. Each image was tiled with 2 × 2 (Tic and 1383D2 lines with long- and short-term exposure to ROCK inhibitor, respectively) and 3 × 3 sections (1383D2 line with long-term exposure to ROCK inhibitor).

### 2.6. Scanning Electron Microscopy (SEM)

For SEM, samples were fixed in phosphate-buffered 2% glutaraldehyde (Electron Microscopy Sciences, Hatfield, PA, USA) in 0.1 M phosphate buffer (PB) and embedded with PB overnight at 4 °C. After the aggregates were fixed, they were post-fixed in 2% osmium tetroxide (Heraeus Chemicals, South Africa) for 2 h on ice. The specimens were then dehydrated in graded ethanol (Nacalai Tesque, Inc., Kyoto, Japan) and dried by t-butyl alcohol freeze-drying (Kanto Chemical Co., Inc., Tokyo, Japan). The dried specimens were coated with 20-nm thick using an osmium plasma ion coater and subjected to SEM observation (S-4800; Hitachi High-Tech America Inc., Schaumburg, IL, USA).

### 2.7. Statistical Analysis

All experiments were conducted in at least thrice to represent the mean with standard deviation. Statistical analysis was performed using unpaired Student’s *t*-test for data comparison between two groups. The Mann-Whitney test was used for data that did not exhibit a normal distribution. All data sets for the distribution of aggregate size were assessed for normality using the Shapiro-Wilk test. Statistical significance was set at *p* < 0.05. 

## 3. Results

### 3.1. Assessment of hiPSC Response in Suspension Culture following Short- and Long-Term Exposure to ROCK Inhibitor

To understand the effect of ROCK inhibitor on morphological and growth characteristics of two lines of hiPSCs in suspension culture, cells were seeded at a density of 2 × 10^2^ cells/well into low-attachment 96-well V-bottom plates using a single-cell passaging method. Figure S1 shows the morphological differences between aggregates from the two different hiPSC lines during short- (24 h) and long-term (120 h) exposure to Y-27632. During short-term exposure, cells formed aggregates within 24 h, and the aggregate size gradually increased until 120 h. However, during long-term exposure, a large spherical cell aggregate was formed over 72 and 120 h. Y-27632 inhibited the collapse of the cell aggregates and resulted in the formation of spherical cell aggregates. In the 1383D2 line subjected to short-term treatment, a spherical cell aggregate was formed after 72 and 120 h. During long-term exposure, large spherical cell aggregates were formed after 72 and 120 h. Large spherical aggregates of the 1383D2 line were larger than small loose aggregates of the Tic line with fewer apoptotic cells, and Y-27632 treatment induced large spherical cell aggregation in both cell lines.

Similar effects were observed for aggregate morphology and growth in treatment groups with two other ROCK inhibitors, Fasudil and Thiazovivin (Figure S1). This suggests that ROCK inhibitor affects aggregate morphology and cell growth in aggregates; however, the effect of ROCK inhibition on long-term cell proliferation has not been examined in detail. Therefore, the effect of Y-27632 in the suspension culture appears to be owing to ROCK inhibition.

### 3.2. The Effect of Long-Term Exposure to ROCK Inhibitor on Growth of hiPSCs

**Figure 1:**
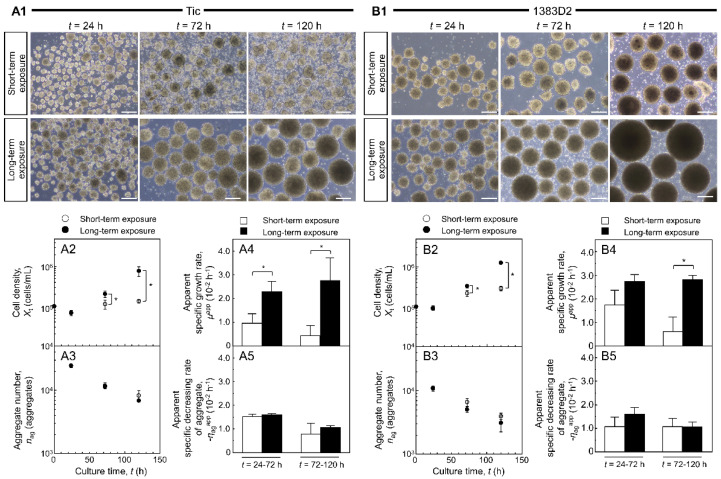
The effect of Rho–associated protein kinase (ROCK) inhibitor on aggregate morphology, cell density, and aggregate number of two different hiPSC lines ((**A**), Tic; (**B**), 1383D2) in suspension culture with short- and long-term exposure to ROCK inhibitor. (**A1**,**B1**) Aggregate morphology. Scale bars, 200 μm. (**A2**–**5**,**B2**–**5**) Cell density, aggregate number, apparent specific growth rate, and apparent specific decreasing rate of aggregates. Statistical significance was analyzed by two-tailed Student’s *t*-tests; * *p* < 0.05 (*n* = 3 per culture). Error bars represent standard deviation. Open circle and bars, short-term exposure to ROCK inhibitor; Closed circle and bars, long-term exposure of ROCK inhibitor.

**Figure 2:**
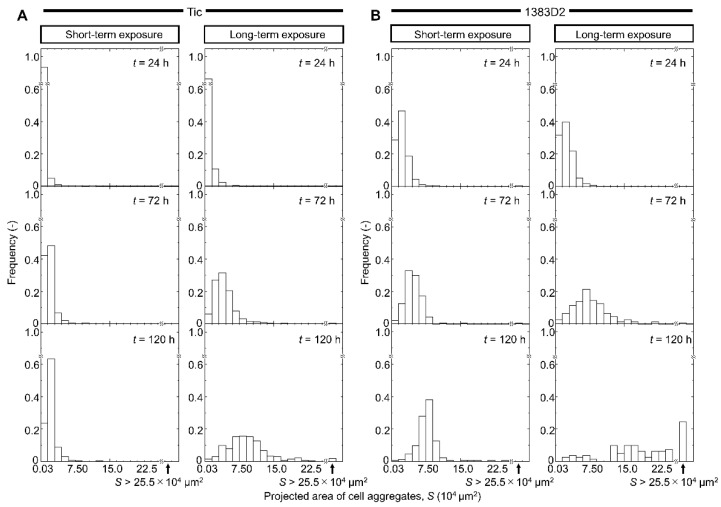
Distribution of projected area of two different hiPSC lines ((**A**), Tic; (**B**), 1383D2) in suspension culture with short- and long-term exposure to ROCK inhibitor at 24, 72, and 120 h. Cell aggregates with 0–300 μm^2^ were not considered as aggregates. All distributions followed non-normal nature based on the Shapiro-Wilk test (*p* < 0.05).

To examine long-term exposure-dependent differences in the growth profiles of the two hiPSC lines in suspension culture, single cells were seeded in a 30-mL bioreactor. Figure 1 shows the differences in aggregate morphology and growth profile of cell aggregates between the two different cell lines during short- and long-term exposure to Y-27632. In all cell lines and conditions, the aggregation of single cells was observed at 24 h, and the size of cell aggregates continuously increased throughout the culture (Figure 2). When aggregate size and morphology were compared to those of short-term exposure, the 1383D2 line formed larger and more spherical aggregates than did the Tic line. For long-term exposure, larger spherical cell aggregates were formed by both cell lines; however, aggregates of the 1383D2 line were much larger than other cell aggregates formed at 120 h.

The apparent specific growth rate (*μ*^app^) of the Tic line was maintained at a lower value than that of the 1383D2 line after short-term exposure. Studies on cell survival and the growth of the two hiPSC lines showed that the formation of small loose aggregates of the Tic line was owing to continued cell death during the exponential phase of growth with single cells extruded from growing hiPSC aggregates. High values of *μ*^app^ were maintained throughout the culture during long-term exposure, and the final cell density was 5.93-fold higher than that during short-term exposure. The *μ*^app^ value of 1383D2 line after short-term exposure gradually decreased throughout the culture, whereas that of cells was continuously maintained throughout the culture during long-term exposure. The final cell density of the long-term exposed culture was 4.17-fold higher than that of the short-term exposed culture. For long-term exposure, higher cell density and no change in aggregate numbers were observed compared to those of aggregates formed after short-term exposure.

Figure 2 shows that the size of aggregate was defined as *S*, and frequency (–) showing the proportion of cell aggregates relative to total aggregates. The size distribution of aggregates was in the range of 0.03 × 10^4^ to 2.55 × 10^5^ μm^2^. Aggregates larger than 2.55 × 10^5^ μm^2^ are represented on the right side of each histogram. In the case of Tic cell lines under short-term exposure, the mode of distribution was maintained 2.25 × 10^4^ μm^2^ at 120 h. Under long-term exposure condition, the mode of distribution at 120 h was higher and wider (8.25 × 10^4^ μm^2^) than that under short-term exposure. ROCK inhibitor induced a comparatively high aggregate size in the Tic line. In 1383D2 line, during short-term exposure to ROCK inhibitor, the mode of distribution increased from 24 to 120 h (8.25 × 10^4^ μm^2^ at 120 h). For long-term exposure, the mode of distribution gradually increased from 2.25 × 10^4^ μm^2^ at 24 h to 6.75 × 10^4^ μm^2^ at 72 h. At 120 h, cell aggregates were present in the range of 1.50 × 10^4^ to 9.00 × 10^4^ μm^2^, and larger aggregates appeared to be evenly distributed in the range of 1.20 × 10^5^ to 2.55 × 10^5^ μm^2^. In addition, much larger aggregates were present than those with a size of 2.55 × 10^5^ μm^2^. The size of aggregates increased, and that of many aggregates more than twice the mode of distribution at 72 h was noticed at 120 h after long-term exposure. In 1383D2 line, right-skewed distribution was formed from 24 h to 72 h, as similar to that in Tic line. For long-term exposure, two flat distributions were formed, while right-skewed distribution was maintained for short-term exposure. Those results indicated it is possible to promote coalescence between cell aggregates by long-term exposure to ROCK inhibitor.

To further confirm that long-term exposure induces coalescence between aggregates, we plated two aggregates during short-and long-term exposure to ROCK inhibitor and assessed their behavior by time-lapse observation. In both hiPSC lines, a faster fusion of aggregates was observed in cultures with long-term exposure (Movie S1).

### 3.3. Localization Pattern of Collagen Type I and pMLC in Cell Aggregates by Prolonged Exposure to ROCK Inhibitor

**Figure 3:**
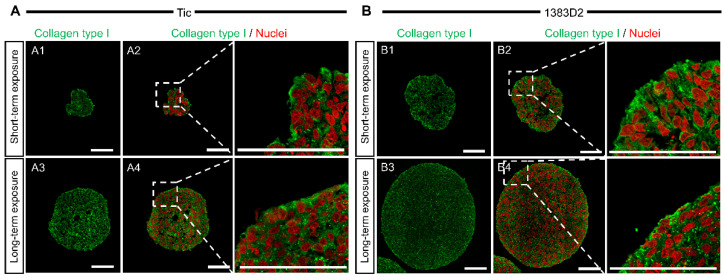
The effect of ROCK inhibitor on collagen type I localization in cell aggregates of two different hiPSC lines ((**A**), Tic; (**B**), 1383D2) under short- and long-term exposure conditions. Florescent images of collagen type I at 120 h showing nuclei (red) and collagen type I (green) (**A1**–**B4**). Scale bars, 100 μm.

Next, we determined whether the enhanced growth of hiPSCs elicited by the ROCK inhibitor was owing to ECM formation. To test this hypothesis, we investigated collagen type I produced by cells to correlate between aggregate formation and collagen type I production in culture with short- and long-term exposure to ROCK inhibitor. Collagen type I expression in the aggregates was evaluated by immunostaining at the culture endpoint (120 h). For short-term exposure, collagen type I was primarily localized at the periphery of hiPSC aggregates regardless of the cell line (Figure 3). When comparing expression differences between the two cell lines, in the 1383D2 line forming large spherical aggregates, collagen type I had a more homogeneous distribution within an aggregate than that in small loose aggregates of the Tic line. For long-term exposure, no change in the expression of collagen type I was observed compared with that for short-term exposure regardless of the cell line. 

**Figure 4:**
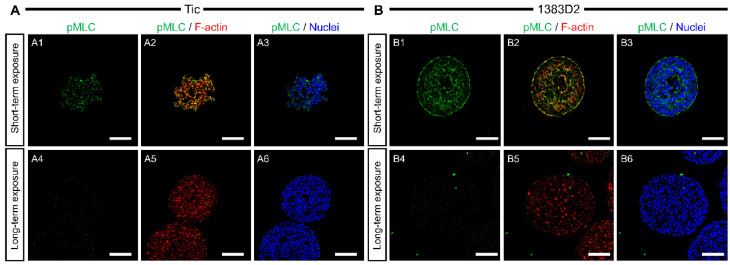
The effect of ROCK inhibitor on phosphorylated myosin (pMLC) and F-actin localization in the aggregates of two different hiPSC lines ((**A**), Tic; (**B**), 1383D2) under short- and long-term exposure conditions. Florescent images of pMLC and F-actin at 120 h showing nuclei (blue), pMLC (green), and F-actin (red) (**A1**–**B6**). Scale bars, 100 μm.

Subsequently, we examined whether long-term exposure to ROCK inhibitor affects the organization of actomyosin cytoskeleton that is responsible for most force-driven processes in cells. We prepared the sections of aggregates stained with pMLC, an active domain of myosin motor proteins, for all cell lines and conditions at 120 h (Figure 4). For short-term exposure, small loose aggregates of Tic cells exhibited stronger pMLC expression at the boundaries between neighboring cells within cell aggregates. In large spherical aggregates of the 1383D2 line, pMLC was found throughout the cells within each cell aggregate. A comparison of the central and peripheral regions of the 1383D2 line aggregates revealed that pMLC was strongly detected at the outer periphery of the cell aggregate. For long-term exposure, pMLC was not detected inside large spherical aggregates in all aggregates of both hiPSC lines. Therefore, the deformation of actomyosin cytoskeleton via long-term ROCK inhibition leads to the survival and enhanced growth of hPSCs in a suspension culture.

### 3.4. Time-Dependent Change of Microstructure on the Surface of Cell Aggregate by Prolonged Exposure to ROCK Inhibitor

**Figure 5:**
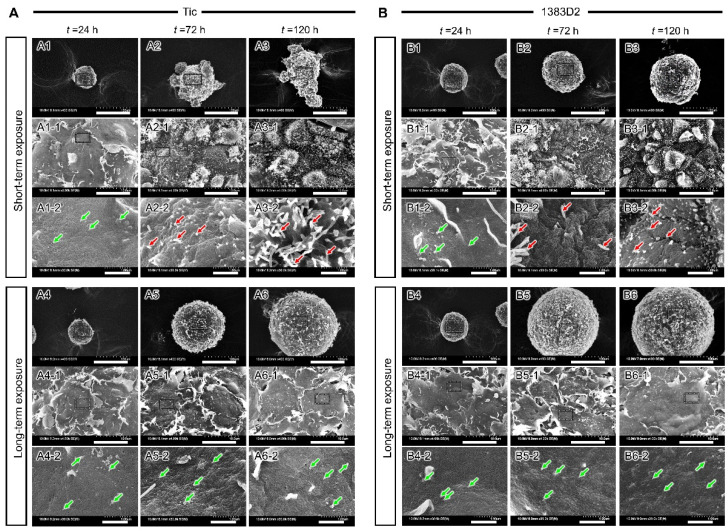
Scanning electron microscopic images showing structural changes in surface of cell aggregates under short- and long-term exposure conditions at 24, 72, and 120 h ((**A**), Tic; (**B**), 1383D2). Panels (**A1-1**–**B6-1**,**A1-2**–**B6-2**) show magnified views of the boxed area. Green arrows and red arrows indicate particle structures and fibril structures, respectively. Scale bars: 100 μm (**A1**–**B6**), 10 μm (**A1-1**–**B6-1**), 1 µm (**A1-2**–**B6-2**).

**Figure 6:**
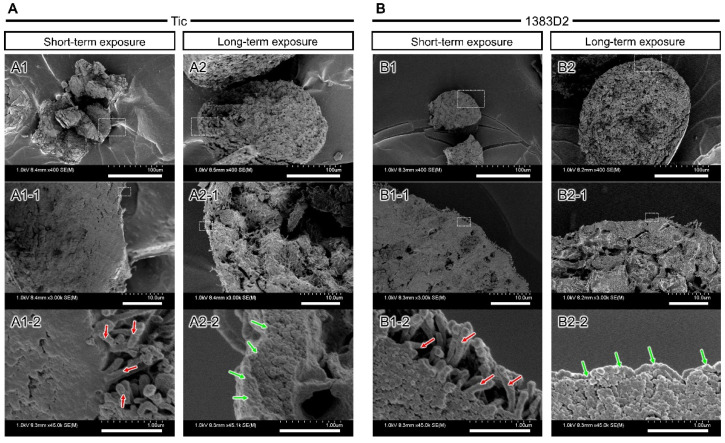
Scanning electron microscopic images of cross-sectional structure of the cell aggregates formed by short- and long-term exposure conditions at 120 h ((**A**), Tic; (**B**), 1383D2). Panels (**A1-1**–**B2-1**,**A1-2**–**B2-2**) show magnified views of the boxed area. Green arrows and red arrows indicate particle structures and fibril structures, respectively. Scale bars: 100 μm (**A1**–**B2**), 10 μm (**A1-1**–**B2-1**), 1 µm (**A1-2**–**B2-2**).

To study the surface microstructures of cell aggregates of two hiPSC lines during short- and long-term exposure to ROCK inhibitor using SEM. Both hiPSC lines formed round-shaped aggregate of approximately 80 μm, with smooth surfaces covered with stacked cells at 24 h. These cells in the aggregates were 10 μm in diameter, and most of them presented a round morphology (Figure 5). When comparing the surface microstructures of cell aggregates between the two hiPSC lines, 1383D2 cells formed spherical aggregates, whereas Tic cells formed loose aggregates with a rough surface. In both hiPSC lines, fibril formation on the uneven surface structure of aggregate was observed at 72 h, and the number of protruding fibril structures increased over time (red arrows). When comparing structural differences between hiPSC lines at the culture endpoint (120 h), in the 1383D2 line forming large spherical aggregates, the fibril structures were shorter than those observed under short-term treatment in the Tic line. When enlarged, particle structures with a diameter of 50–200 nm adhered to each other were noticed. Some fibril structures existed in those structures (red arrows). Those fibril structures with a diameter of approximately 100 nm and a stripe of approximately 50 nm were observed. The cross-sectional structure of the cell aggregate was flat, and the inside of the cell aggregate had a dense structure similar to that of the Tic line (Figure 6). When enlarged, a fibril structure was vertically produced from the surface of the cell (red arrows). For long-term exposure, an increase in size of the fibrillar structure on surface of aggregates was inhibited regardless of hiPSC lines (Figure 5). The enlarged SEM images represent particle structures on the surface of aggregates at 72 and 120 h (green arrows). In the cross-sectional structure of cell aggregates, the intercellular space was larger at 120 h (Figure 6). When enlarged, particle structures existed on the surface of the cell aggregates and were bound to each other (green arrows). These results suggest that cell-secreted particle structures were maintained on the surface of cell aggregates, and the intercellular space was larger for a cell aggregate by long-term exposure to ROCK inhibitor in both cell lines.

## 4. Discussion

**Figure 7:**
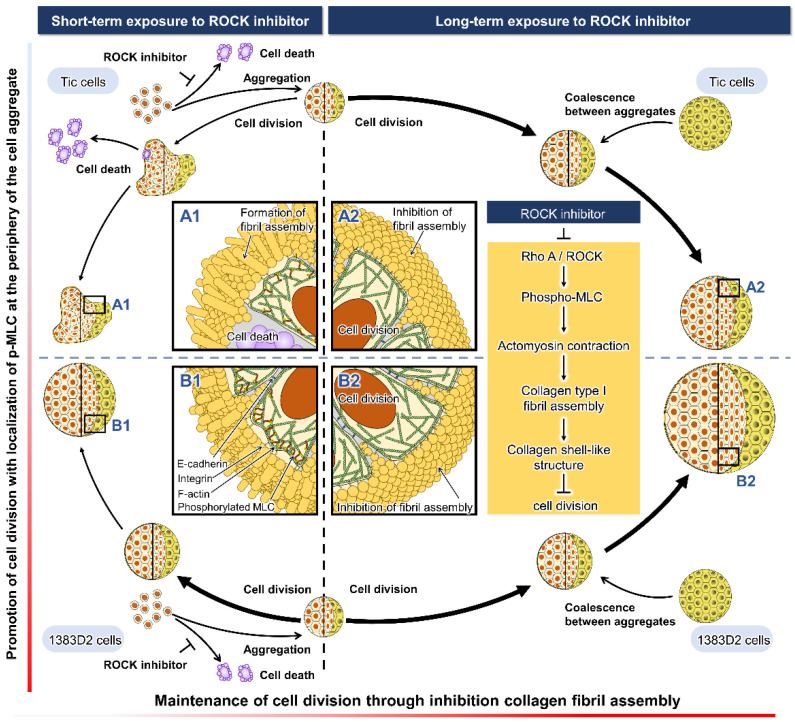
Schematic drawing of our hypothesis that cell-derived collagen type I regulates cell division and coalescence between aggregates of two different hiPSC lines ((**A**), Tic; (**B**), 1383D2) by inhibiting actomyosin contraction in suspension culture with prolonged ROCK inhibition. Panels (**A1**–**B2**) show magnified views of the boxed areas, respectively.

Although several beneficial treatments with ROCK inhibitors have been reported for cell growth [14,15,16,17,18,19], no studies investigated the long-term effects on the growth behavior of hiPSCs in suspension culture. The aim of this study was to understand the process of cell growth in hiPSC aggregates by clarifying the long-term effects of ROCK inhibitors on growth behavior in suspension culture. We found that the structural alteration of the ECM played a key role in growth behaviors for aggregate formation, such as cell division, collapse, and coalescence between aggregates under suspension culture conditions. The process of aggregate formation in two contrasting hiPSC lines that may easily form aggregates was investigated using a suspension culture system (Figure 7). 

When the aggregate morphology and size were compared throughout the culture (Figure 1), the 1383D2 line formed large and spherical aggregates (Figure 7B) as compared to those of the Tic line (Figure 7A). Furthermore, the apparent specific growth rate of the 1383D2 line was higher than that of the Tic line. The cell aggregates of the Tic line were deformed and easily collapsed because they were more sensitive to liquid flow than the 1383D2 line (Figure 2). These morphological changes in cell aggregates showed that actomyosin contractility was affected by ECM disruption induced by fluid flow, resulting in an altered ability to form aggregates [24,25]. Phosphorylated MLC regulates mechanical properties of the cytoskeleton and significantly alters cell growth [26]. Therefore, cell aggregate formation and cell growth are related to the cytoskeleton of hiPSCs and the environment in which they are formed in a suspension culture. Immunostaining of the Tic lines indicated pMLC localization of cells inside aggregates (Figure 4A), whereas pMLC was localized strongly at the periphery of aggregates of the 1383D2 line (Figure 4B). Furthermore, highly dense collagen type I was localized at the outer part of peripheral cells in the 1383D2 line (Figure 3B). ECM proteins within the cell aggregate act as biomechanical signaling molecules and exhibit biomechanical roles in influencing the balance of tension between the cell cytoskeleton and the extracellular microenvironment [27,28]. This may be related to cell adhesion molecules that mediate cell-cell and cell-substrate adhesion. Such adhesion promotes actin organization and generates mechanical forces that constitute the morphology of cell aggregate. We previously found that hiPSCs with low capacity of ECM secretion, such as collagen type I, had a high frequency of apoptotic cells detached from the peripheral region of cell aggregates [5]. Stable aggregate formation and cell growth in aggregates may indicate the presence of active tensional homeostasis [22,27,28]. Through the ECM and other cells in the peripheral regions of the aggregate, cells may modify the surrounding environment similar to their own characteristic phenotype [27,28]. These changes are expected to increase the forces generated at the sites of cytoskeleton binding to the ECM and adjacent cells. Although the 1383D2 cell line can stably form aggregates compared to that of Tic cell lines, the apparent specific growth rate of the 1383D2 line decreased in the late phase of the suspension culture. cell division in aggregates decreases in the late phase of culture by the accumulation of cell-secreted ECM [6,7]. In this study, collagen type I and pMLC were localized at the peripheral region of the cell aggregates. Large spherical cell aggregates were stably formed by actomyosin-generated contraction in suspension culture, so that a collagen type I shell-like structure may be formed at the surface of the cell aggregate. Consistent with previous studies [29,30], this hypothesis was supported by the observation of fibril structures on the surface of cell aggregates. However, the formation of collagen type I shell-like structures induces cell death by restricting the diffusion of nutrients and oxygen into the center of cell aggregates [6,30]. Therefore, a culture method that enables more stable aggregate formation and cell growth is required because cell growth is reduced in the late phase. These findings provide important information for understanding the growth behavior of hiPSCs in suspension cultures. This suggests that the interaction between adjacent cells and the ECM by changing the cytoskeleton is closely related to stable aggregate formation and enhancement of cell division in aggregates during the growth phase of cell aggregates. 

Long-term exposure to ROCK inhibitor resulted in different growth kinetics of the two hiPSC lines in terms of cell division and coalescence between cell aggregates based on the morphology and size of the aggregates. In both hiPSC lines, aggregates by long-term exposure to ROCK inhibitor showed higher expansion fold and apparent specific growth rate compared to those of aggregates by short-term exposure. Furthermore, the apparent specific growth rate of the 1383D2 line by short-term exposure gradually decreased in the late phase, whereas that of cells by long-term exposure was continuously maintained throughout the culture. In this study, we hypothesized that the maintenance of ECM structure consequent to inhibition of actomyosin contractility by ROCK inhibitor leads to sustained growth throughout the suspension culture of hiPSCs. Long-term exposure to ROCK inhibitor induces the stable cell aggregate formation in the Tic cell line, which is easily collapsed by mechanical stress. ROCK inhibitors have been shown to regulate cell proliferation and differentiation by activating the extracellular signal-regulated kinase (ERK) pathway [18,19]. ROCK inhibitors also promote cell survival by suppressing cell death of single hiPSCs through the p53 pathway [10,11,12,31,32,33]. Therefore, ROCK inhibitors can provide stable cell division by regulating cell signaling pathways. In addition, ROCK inhibitors may control colony morphology, aggregate compaction, and pluripotency by regulating ROCK-myosin pathway-dependent mechanical stress-induced actomyosin contraction [34]. The contractility of actomyosin influences the shapes of cells and nucleus [35]. Consistent with a previous study [36], our observations support the hypothesis that actomyosin contractility affects the shape and integrity of nuclei in aggregates and changes biochemical signals in the cell. Therefore, ROCK inhibition may affect cell division not only directly by biochemical signals but also indirectly by changes in cell morphology through actomyosin contraction and cell adhesion. We observed that long-term ROCK inhibition enhanced the cell growth of hiPSC aggregates by blocking the structural alteration on the surface of cell aggregates. Immunofluorescence staining revealed spatial heterogeneity of phosphorylated MLC inside the aggregates at their periphery after short-term exposure (Figure 7(A1,B1)), whereas aggregates by long-term exposure did not localize it within cell aggregates (Figure 7(A2,B2)). The level of phosphorylated MLC increases by ROCK [37]. The actomyosin cytoskeleton, induced by phosphorylated MLC, generates contractile or tensile forces in individual cells. The particle structure of collagen type I generated by inhibiting actomyosin contraction may suppress the formation of collagen shell-like structure because the fibril structure of collagen type I is formed by actomyosin-induced plasma membrane contraction [38]. Therefore, the maintenance of the ECM structure by inhibition of actomyosin contraction may be related to the maintenance of cell division by inhibiting the formation of collagen type I shell-like structures. However, further consideration will be needed to yield any findings about the nature structure and function of particle structure since it is not clear whether the particle structure is collagen type I. Furthermore, while long-term ROCK inhibition increased the average aggregate size, the number of distinct hiPSC aggregates decreased as adjacent aggregates coalesced. Cell division is decreased by the formation of larger cell aggregates via coalescence between cell aggregates [6]. ROCK inhibitors may maintain cell division by blocking the Hippo pathway through the cell contact inhibition within cell aggregates. Therefore, downregulation of the ROCK pathway by the ROCK inhibition enhanced stable aggregate formation and maintained the division of hiPSCs in suspension culture with maintenance of collagen type I structure and without pMLC localization. These findings contribute to the development of culture processes of hiPSCs in suspension culture, suggesting that the regulation of ECM structure through pMLC localization is closely related to cell growth during the growth phase in aggregate.

## 5. Conclusions

In conclusion, we demonstrated that ROCK inhibition promoted cell division related to ECM structural alterations of hiPSCs in suspension culture. The effects on cell division could be explained by the maintenance of ECM structure through inhibition of pMLC within an aggregate after long-term exposure to ROCK inhibitors. Therefore, an understanding of the structural differences in the ECM between the two hiPSC lines will facilitate the understanding growth behavior of hiPSC aggregates after cell aggregation in suspension culture. 

## Data Availability

The data presented in this study are available on request from the corresponding author.

## Supplementary Materials

The following supporting information can be downloaded at: https://www.mdpi.com/article/10.3390/bioengineering9110613/s1, Figure S1, The effect of ROCK inhibition on aggregate morphology of different cell lines (Tic and 1383D2). The aggregates were cultured with ROCK inhibitors Y-27632(A–D), Fasudil (E–H), and Thiazovivin (I–L). Scale bars, 200 μm; Movie S1, Time-lapse images of coalescence between hiPSC aggregates (Tic and 1383D2 cell lines) in static suspension culture under short- and long-term exposure to ROCK inhibitor at 72 h. Scale bars, 200 μm.
