# Supplementary figures and images for: Effect of Rho–Associated Kinase Inhibitor on Growth Behaviors of Human Induced Pluripotent Stem Cells in Suspension Culture
Source: Bioengineering (Basel). 2022 Oct 25;9(11):613. doi: 10.3390/bioengineering9110613 (PMC9687832; doi:10.3390/bioengineering9110613)

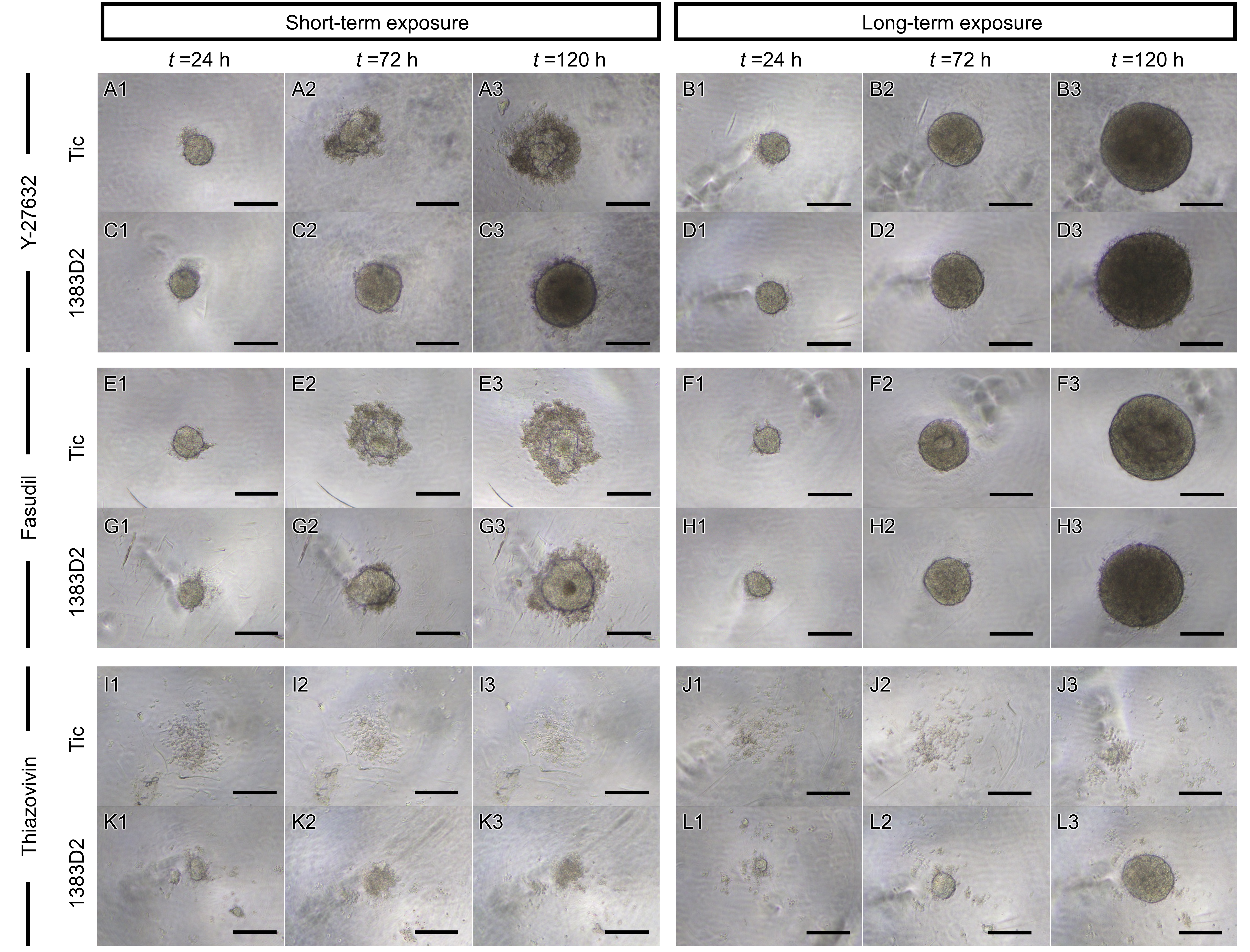

Supplement: Supplementary file 1 [file bioengineering-09-00613-s001.zip › Supplementary File/Figure S1.tif]
